# Sensitivity of different epidermal growth factor receptor (EGFR) exon 19 deletion subtypes to first-line osimertinib in Chinese non-small cell lung cancer patients

**DOI:** 10.1186/s12885-026-15908-4

**Published:** 2026-03-30

**Authors:** Dongming Zhang, Yuequan Shi, Xiaoyan Liu, Xiaoxing Gao, Minjiang Chen, Jing Zhao, Wei Zhong, Shengjie Li, Yan Xu, Mengzhao Wang

**Affiliations:** 1 Department of Respiratory and Critical Care Medicine, Peking Union Medical College Hospital, Chinese Academy of Medical Sciences & Peking Union Medical College, No. 1 Shuaifuyuan Wangfujing, Dongcheng District, Beijing, 100730 China; 2 Department of Internal Medicine, Peking Union Medical College Hospital, Chinese Academy of Medical Sciences & Peking Union Medical College, Beijing, 100730 China; 3 Biomedical Engineering Facility of National Infrastructures for Translational Medicine, Institute of Clinical Medicine, Peking Union Medical College Hospital, Chinese Academy of Medical Sciences and Peking Union Medical College, Beijing, 100730 China

**Keywords:** EGFR, Exon 19 deletion, Non-small cell lung cancer, Osimertinib

## Abstract

**Background:**

Research on the efficacy of first-line osimertinib in non-small cell lung cancer (NSCLC) patients with different epidermal growth factor receptor (EGFR) exon 19 deletion (19del) mutation sites is limited. This study aimed to evaluate whether different EGFR 19del subtypes are associated with differential clinical outcomes in patients with EGFR-mutant NSCLC receiving first-line osimertinib therapy.

**Methods:**

This study included 106 NSCLC patients with stage IIIb–IV disease from the CAPTRA-Lung database, confirmed to have EGFR 19del mutations through next-generation sequencing (NGS) and receiving first-line osimertinib. Patients were categorized based on mutation frequencies (common E746_A750del mutation or other uncommon subtypes), the deletion start codon for the EGFR 19del (E746 or L747), and the number of deleted nucleotides (15-nucleotide deletion [15n-del] or non-15n-del). Clinical characteristics, objective response rate (ORR) and progression-free survival (PFS) were compared between these subgroups.

**Results:**

A total of 19 EGFR 19del mutation subtypes were identified. The most frequent mutation site for exon 19 deletion was E746_A750del, accounting for 56.6% of cases (*n* = 60), followed by L747_P753delinsS (9.4%, *n* = 10), L747_T751delinsP (6.6%, *n* = 7), and L747_A750delinsP (4.7%, *n* = 5). Patients in the non-15n-del group had a higher prevalence of baseline brain metastases compared with those in the 15n-del group (41.7% vs. 22.9%; *P* = 0.044). No associations were found between ORR and PFS of first-line osimertinib treatment and mutation frequencies, the deletion start codon for the EGFR 19del, and the number of deleted nucleotides. However, certain subtypes, particularly L747_A750delinsP, showed a trend toward a shorter PFS compared with the common E746_A750del subtype, although these differences did not reach statistical significance (12.3 months vs. 24.3 months; *P* = 0.100).

**Conclusions:**

Overall, EGFR 19del subtypes showed comparable responses to first-line osimertinib. However, the trend toward poorer outcomes in the L747_A750delinsP subtype suggests potential heterogeneity that warrants confirmation in larger cohorts with longer follow-up.

## Background

Epidermal growth factor receptor (EGFR) mutations occur in approximately 50% of Asian patients with non-small cell lung cancer (NSCLC), with exon 19 deletion (19del) and exon 21 L858R being the most common subtypes [1, 2]. These mutations correlate with a favorable response to EGFR-tyrosine kinase inhibitors (EGFR-TKIs). Due to structural differences, EGFR 19del confers higher affinity for EGFR-TKIs and more potent downstream signaling inhibition compared with the L858R mutation. Additionally, the frequency of co-mutations is lower in EGFR 19del, contributing to better responses to EGFR-TKIs [3–6]. In the FLAURA study, first-line osimertinib demonstrated a median progression-free survival (PFS) of 21.4 months for EGFR 19del mutations and 14.4 months for L858R mutations [7]. This highlights the higher sensitivity of EGFR 19del mutations to EGFR-TKIs compared to L858R mutations.

Furthermore, different EGFR 19del subtypes may be associated with heterogeneous responses to EGFR-TKIs. Utilizing next-generation sequencing (NGS), various subtypes of EGFR 19del mutations are identified, with E746_L746del being the most common according to the Catalogue of Somatic Mutations in Cancer (COSMIC) database [8, 9]. While numerous studies have explored differences in sensitivity to first- or second-generation EGFR-TKIs among EGFR 19del mutation sites, the results are controversial and research addressing the efficacy of first-line osimertinib across these sites is limited [6, 10–25]. Given osimertinib’s approval and widespread use in first-line treatment for advanced NSCLC patients with EGFR mutations, investigating the sensitivity of different EGFR 19del subtypes to first-line osimertinib becomes imperative.

Therefore, this multicenter retrospective study aimed to evaluate whether different EGFR 19del subtypes, classified by mutation frequency (common E746_A750del vs. others), deletion start codon (E746 vs. L747), and deletion length (15n-del vs. non-15n-del), are associated with differential efficacy of first-line osimertinib in patients with EGFR-mutant NSCLC.

## Methods

### Patients

The CAPTRA-Lung study (NCT03334864), led by Peking Union Medical College Hospital, is a multicenter, real-world registry dedicated to collecting data from Chinese patients with advanced or metastatic NSCLC [26]. Over 30 research centers actively participate in this study, contributing valuable insights from diverse clinical settings. Our multicenter, retrospective cohort study focused on exploring the sensitivity of different EGFR 19del subtypes to first-line osimertinib and collected patients from the CAPTRA-Lung database.

The primary inclusion criteria for this study were as follows: (1) diagnosis of stage IIIb-IV NSCLC according to the 8th edition of the American Joint Committee on Cancer (AJCC) TNM staging system between June 2017 and August 2023; (2) confirmation of EGFR 19del mutation through NGS; (3) initiation of first-line treatment with osimertinib; (4) availability of relatively complete baseline characteristics and follow-up data. Exclusion criteria included: (1) missing genetic testing reports or an inability to specify the EGFR 19del subtype; (2) first-line treatment with first- or second-generation EGFR-TKIs; (3) incomplete clinical information data. Patients were stratified based on mutation frequencies (common E746_A750del mutation or other uncommon subtypes), the deletion start codon for EGFR 19del (E746 or L747), and the number of deleted nucleotides (deletions of 15 nucleotides [15n-del] or non-15n-del).

### Data collection

The primary outcomes of the study were PFS and objective response rate (ORR). Clinical variables, including gender, age, Eastern Cooperative Oncology Group (ECOG) performance status, smoking history, tumor history, family history of tumors, pathological type, staging, metastasis status, and treatment outcomes, were extracted from hospital case records. Treatment efficacy was assessed based on the Response Evaluation Criteria in Solid Tumours (RECIST) version 1.1, categorizing responses as complete response (CR), partial response (PR), stable disease (SD), or progressive disease (PD). ORR was defined as the proportion of patients achieving CR or PR. PFS was calculated from the initiation of treatment to the onset of disease progression. Follow-up assessments were conducted every three months until disease progression, with the last follow-up performed on August 1, 2024.

### Statistical analysis

Statistical analyses were carried out using SPSS version 22.0 (IBM Corp., Armonk, NY). Group comparisons for categorical variables utilized either the chi-square test or Fisher’s exact test, with statistical significance set at a two-sided P-value less than 0.05. Survival analysis for PFS employed the Kaplan-Meier (KM) method, and the log-rank test was applied. The Cox proportional hazards regression model was utilized for both univariate and multivariate analyses. Graphs were generated using GraphPad Prism 8.0 (San Diego, CA, USA) and R software (version 4.1.1, R Foundation for Statistical Computing, Vienna, Austria).

## Results

### Patient characteristics

From June 2017 to August 2023, a total of 106 NSCLC patients who received first-line osimertinib were ultimately included in this study from the CAPTRA-Lung database, spanning six research centers. The clinical characteristics are summarized in Table 1. The median age was 62 years (range, 26–86 years). The majority of patients were female (59.4%), had an ECOG performance status of 0–1 (94.3%), and were non-smokers (73.6%). A history of prior tumors was present in 4.7% of patients, and 19.8% had a family history of cancer. Adenocarcinoma constituted the majority (97.2%). Most patients were in TNM stage IVa-IVb (91.5%) with 0–3 distant organ metastases (89.6%). At the time of diagnosis, bone metastasis, liver metastasis, and brain metastasis were present in 49.1%, 11.3%, and 29.2% of patients, respectively.

**Table 1:** Baseline characteristics of patients and treatment responses with EGFR 19del variants

Characteristics	All patients	E746_A750del	Others	*P* value	E746	L747	*P* value	15n-del	Non-15n-del	*P* value
	(*N* = 106)	(*N* = 60)	(*N* = 46)		(*n* = 69)	(*n* = 33)		(*n* = 70)	(*n* = 36)	
Age (years)				0.398			0.99			0.125
Median (range)	62 (26–86)									
≤65 years	69 (65.1%)	37 (61.7%)	32 (69.6%)		44 (63.8%)	21 (63.6%)		42 (60%)	27 (75%)	
>65 years	37 (34.9%)	23 (38.3%)	14 (30.4%)		25 (36.2%)	12 (36.4%)		28 (40%)	9 (25%)	
Gender				0.183			0.751			0.066
Female	63 (59.4%)	39 (65%)	24 (52.2%)		42 (60.9%)	19 (57.6%)		46 (65.7%)	17 (47.2%)	
Male	43 (40.6%)	21 (35%)	22 (47.8%)		27 (39.1%)	14 (42.4%)		24 (34.3%)	19 (52.8%)	
ECOG				0.349			0.691			0.172
0–1	100 (94.3%)	55 (91.7%)	45 (97.8%)		64 (92.8%)	32 (97%)		64 (91.4%)	36 (100%)	
2–3	6 (5.7%)	5 (8.3%)	1 (2.2%)		5 (7.2%)	1 (3%)		6 (8.6%)	0 (0%)	
Smoking history				0.087			0.544			0.104
No	78 (73.6%)	48 (80%)	30 (65.2%)		52 (75.4%)	23 (69.7%)		55 (78.6%)	23 (63.9%)	
Yes	28 (26.4%)	12 (20%)	16 (34.8%)		17 (24.6%)	10 (30.3%)		15 (21.4%)	13 (36.1%)	
Tumor history				0.76			1			1
No	101 (95.3%)	58 (96.7%)	43 (93.5%)		66 (95.7%)	32 (97%)		67 (95.7%)	34 (94.4%)	
Yes	5 (4.7%)	2 (3.3%)	3 (6.5%)		3 (4.3%)	1 (3%)		3 (4.3%)	2 (5.6%)	
Family history of tumor				0.584			0.348			0.655
No	85 (80.2%)	47 (78.3%)	38 (82.6%)		53 (76.8%)	28 (84.8%)		57 (81.4%)	28 (77.8%)	
Yes	21 (19.8%)	13 (21.7%)	8 (17.4%)		16 (23.2%)	5 (15.2%)		13 (18.6%)	8 (22.2%)	
Histological types				0.815			1			0.521
Adenocarcinoma	103 (97.2%)	59 (98.3%)	44 (95.7%)		67 (97.1%)	32 (97%)		67 (95.7%)	36 (100%)	
Others	3 (2.8%)	1 (1.7%)	2 (4.3%)		2 (2.9%)	1 (3%)		3 (4.3%)	0 (0%)	
Stage				1			1			1
Ⅲb-Ⅲc	9 (8.5%)	5 (8.3%)	4 (8.7%)		6 (8.7%)	3 (9.1%)		6 (8.6%)	3 (8.3%)	
Ⅳa-Ⅳb	97 (91.5%)	55 (91.7%)	42 (91.3%)		63 (91.3%)	30 (90.9%)		64 (91.4%)	33 (91.7%)	
Number of metastatic sites				0.144			0.16			0.406
0–3	95 (89.6%)	51 (85%)	44 (95.7%)		59 (85.5%)	32 (97%)		61 (87.1%)	34 (94.4%)	
≥4	11 (10.4%)	9 (15%)	2 (4.3%)		10 (14.5%)	1 (3%)		9 (12.9%)	2 (5.6%)	
Bone metastasis				0.865			0.94			0.583
No	54 (50.9%)	31 (51.7%)	23 (50%)		35 (50.7%)	17 (51.5%)		37 (52.9%)	17 (47.2%)	
Yes	52 (49.1%)	29 (48.3%)	23 (50%)		34 (49.3%)	16 (48.5%)		33 (47.1%)	19 (52.8%)	
Liver metastasis				0.172			0.802			0.308
No	94 (88.7%)	51 (85%)	43 (93.5%)		60 (87%)	30 (90.9%)		60 (85.7%)	34 (94.4%)	
Yes	12 (11.3%)	9 (15%)	3 (6.5%)		9 (13%)	3 (9.1%)		10 (14.3%)	2 (5.6%)	
Brain metastasis				0.126			0.655			0.044
No	75 (70.8%)	46 (76.7%)	29 (63%)		51 (73.9%)	23 (69.7%)		54 (77.1%)	21 (58.3%)	
Yes	31 (29.2%)	14 (23.3%)	17 (37%)		18 (26.1%)	10 (30.3%)		16 (22.9%)	15 (41.7%)	
Osimertinib best response				0.819			0.359			0.780
CR	1 (0.9%)	1 (1.7%)	0 (0%)		1 (1.4%)	0 (0%)		1 (1.4%)	0 (0%)	
PR	69 (65.1%)	40 (66.7%)	29 (63%)		46 (66.7%)	19 (57.6%)		44 (62.9%)	25 (69.4%)	
SD	36 (34.0%)	19 (31.7%)	17 (37%)		22 (31.9%)	14 (42.4%)		25 (35.7%)	11 (30.6%)	
ORR	70 (66.0%)	41 (68.3%)	29 (63%)	0.569	47 (68.1%)	19 (57.6%)	0.198	45 (64.3%)	25 (69.4%)	0.595

*ECOG *Eastern cooperative oncology group, *CR *Complete response, *PR *Partial response, *SD *Stable disease, *ORR *Objective response rate, *E746 *Deletions starting at E746, *L747 *Deletions starting at L747, *15n-del *Deletions of 15 nucleotides, *non-15n-del* non-15 nucleotide deletion

There were no significant differences in baseline characteristics between the common E746_A750del group and other uncommon mutation groups, or between the deletion start codon being E746 and L747 groups. However, in the non-15n-del group, 41.7% of patients had baseline brain metastasis, significantly higher than the 22.9% in patients with 15n-del (*P* = 0.044).

### EGFR 19del subtypes

**Fig. 1:**
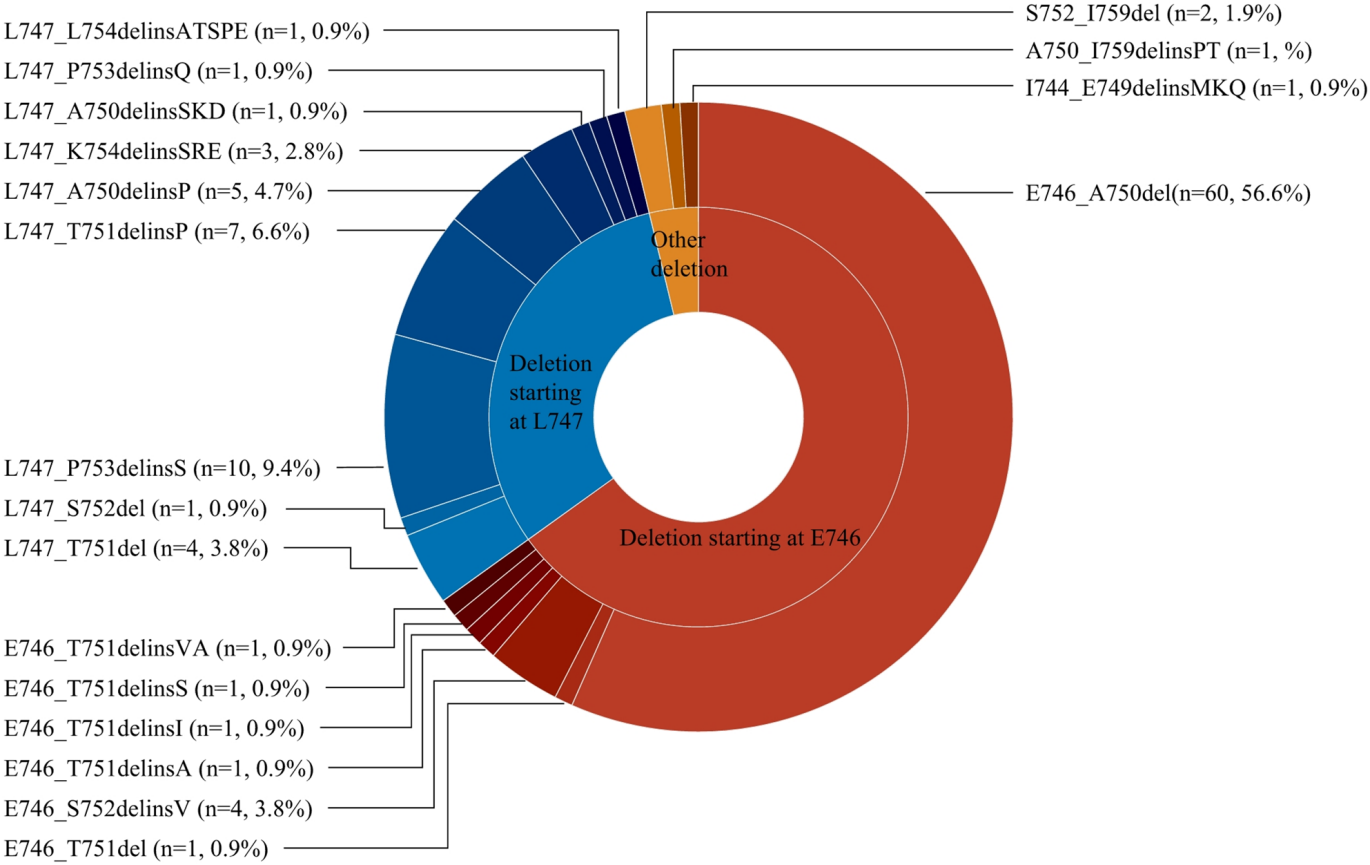
Distribution of EGFR exon 19 deletion subtypes. The pie chart shows the frequency of each subtype in the study cohort (*N* = 106)

A total of 19 EGFR 19del mutation subtypes were identified (Fig. 1). The most common variant was E746_A750del, accounting for 56.6% of cases (*n* = 60), followed by L747_P753delinsS (9.4%, *n* = 10), L747_T751delinsP (6.6%, *n* = 7), and L747_A750delinsP (4.7%, *n* = 5). The deletion start codon at E746 and L747 was observed in 65.1% (*n* = 69) and 31.1% (*n* = 33) of cases, respectively. There were 70 patients (66.0%) with 15n-del and 36 patients (34.0%) with deletions greater or less than 15 nucleotides.

### Sites of disease progression

**Fig. 2:**
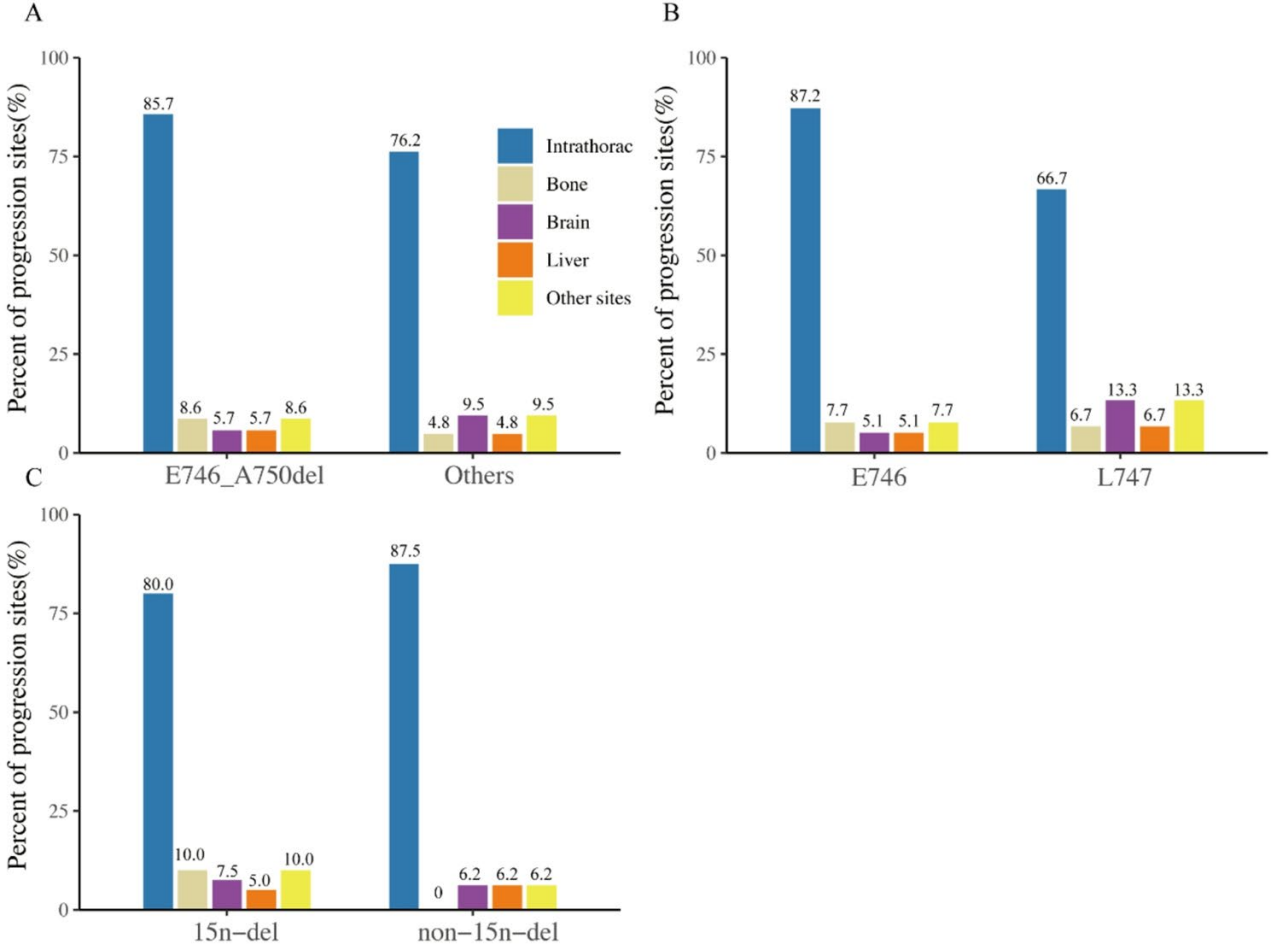
Sites of disease progression in patients with different EGFR 19del subtypes. The bar chart illustrates the distribution of progression sites among patients who experienced disease progression during follow-up

During follow-up, a total of 56 patients experienced disease progression after treatment with osimertinib. We analyzed the sites of disease progression among different groups (Fig. 2). Intrathoracic metastases were the most common metastases in the E746_A750del group, accounting for 85.7% of the metastases in patients, followed by 8.6% in the bone, 8.6% in the brain, and only 5.7% in the liver. Similar to the E746_A750del group, the most common metastases in the uncommon mutation group were also intrathoracic metastases (76.2% of patients). The proportion of brain metastases was numerically higher in the uncommon mutation group than that in the E746_A750del group (9.5% vs. 5.7%, *P* > 0.99), and the rate of bone metastases was lower than that in the E746_A750del group (4.8% vs. 8.6%, *P* > 0.99). In addition, no associations were found between sites of disease progression and the deletion start codon for EGFR 19del, and the number of deleted nucleotides.

### Outcomes of EGFR 19del patients

As of August 1, 2024, the median follow-up duration for all patients was 23.8 months (95% confidence interval [CI]: 21.7–25.9 months). All patients achieved disease control, with 1 (0.9%) achieving CR, 69 (65.1%) having PR, and 36 (34.0%) exhibiting SD (Table 1). None of the patients had progressive disease as the best treatment response. The overall ORR was 66.0%. In the common mutation group (E746_A750del) and other groups, the ORR was 68.3% and 63.0%, respectively (*P* = 0.569). Additionally, ORR demonstrated no significant differences between the deletion start codon being E746 group and L747 groups, or between the 15n-del and non-15n-del groups (68.1% vs. 57.6%, *P* = 0.198; 64.3% vs. 69.4%, *P* = 0.595).

**Fig. 3:**
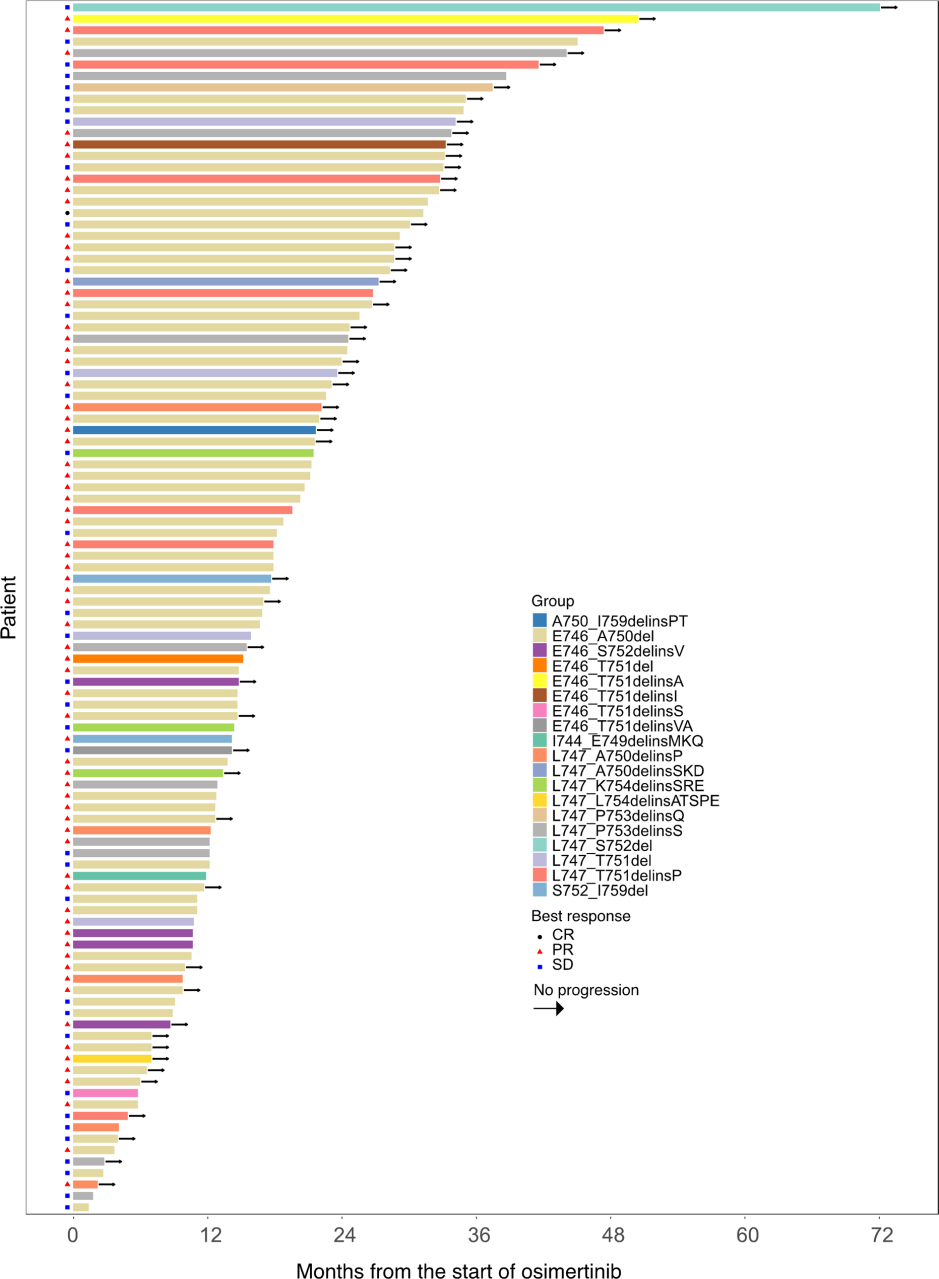
Swimmer plot of progression-free survival. Each bar represents an individual patient (*N* = 106), with bar length indicating PFS duration. Arrows denote patients continuing treatment at the data cutoff (August 1, 2024)

**Fig. 4:**
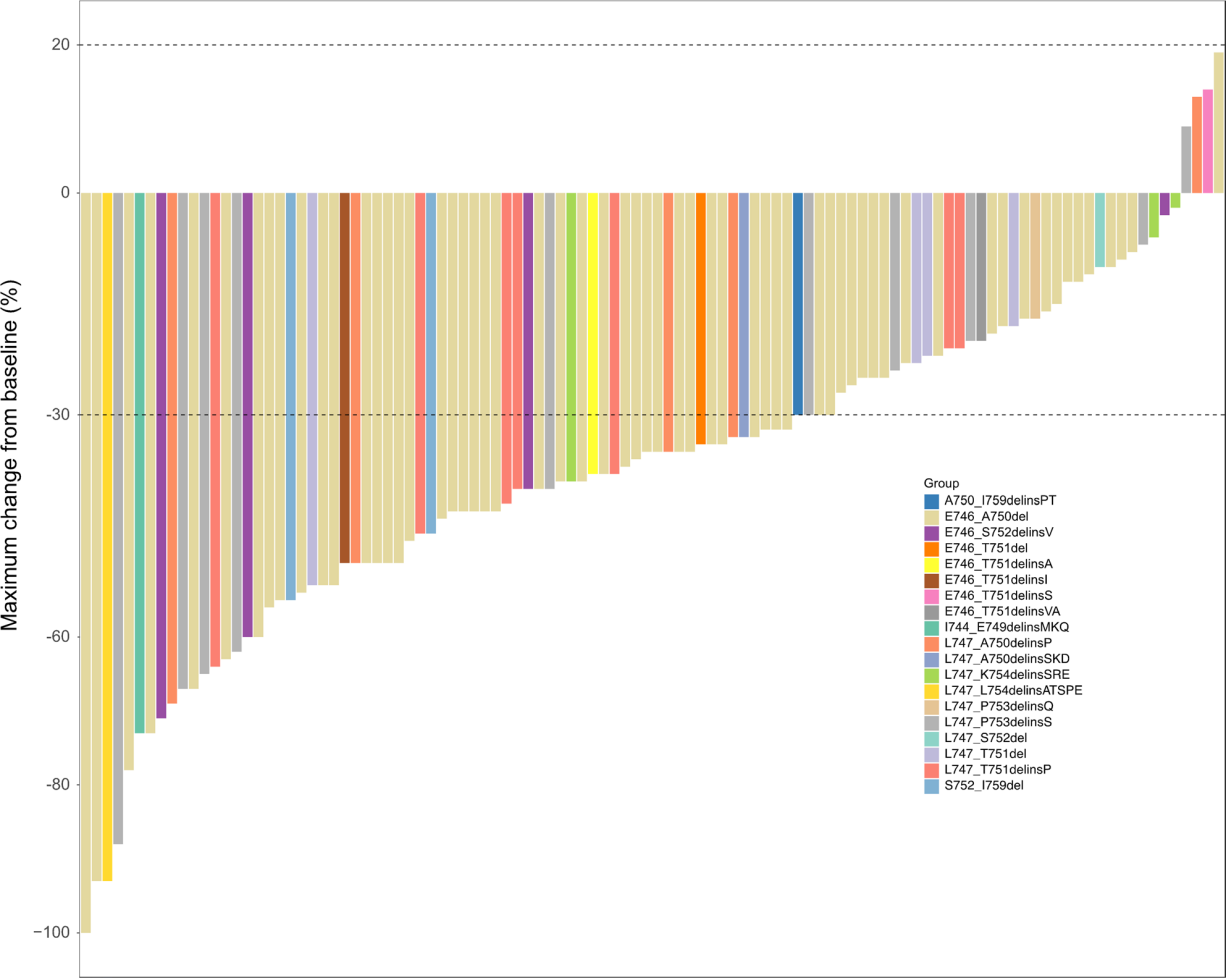
Waterfall plot of best objective response. Each bar represents the maximum percentage change in target lesion size from baseline in individual patients (*N* = 106). The dashed lines indicate thresholds for partial response (− 30%) and progressive disease (+ 20%)

**Fig. 5:**
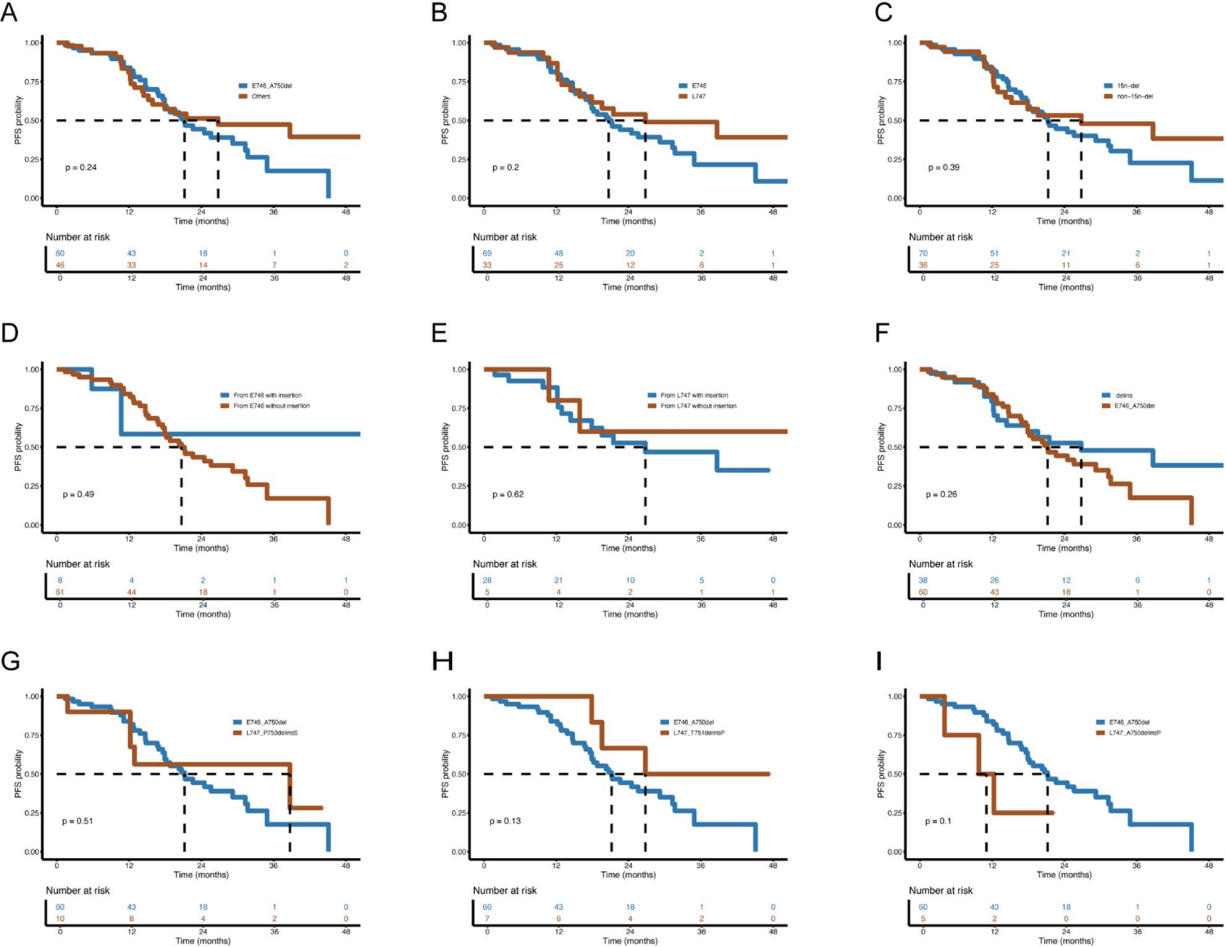
Kaplan–Meier curves of progression-free survival according to EGFR 19del subtypes. Patients were stratified by (**A**) mutation frequency (E746_A750del vs. others), (**B**) deletion start codon (E746 vs. L747), and (**C**) deletion length (15n-del vs. non-15n-del). P-values were calculated using the log-rank test

The median PFS for all patients was 21.3 months (95% CI: 15.3–27.3 months). The swimmer plot for PFS is illustrated in Fig. 3. The maximum change in target lesion size is shown in Fig. 4. As depicted in Fig. 5, the median PFS for E746_A750del was 21.2 months (95% CI: 16.3–26.1), shorter than other uncommon EGFR 19del mutations (26.8 months, 95% CI: 7.5–46.1), but without statistical significance (*P* = 0.245). No significant difference was observed in median PFS between deletion starting codon at E746 and L747 group (20.7 months [95% CI: 15.8–25.6] vs. 26.8 months [95% CI: 7.0-46.6], *P* = 0.201). For patients in the 15n-del group, the median PFS was 21.3 months (95% CI: 16.8–25.8), shorter than those in the non-15n-del group (26.8 months [95% CI: 5.8–47.8]) (*P* = 0.386).

Additionally, for patients with deletions starting at E746, the median PFS for those with insertion was not reached, longer than those without insertion mutations at 20.7 months (95% CI: 15.9–25.5), but without statistical significance (*P* = 0.488). For patients with deletions starting at L747, the median PFS for those with insertion was 26.8 months (95% CI: 8.6–45.0 months), while the median PFS for those without insertion has not been reached (*P* = 0.620). Furthermore, patients with E746_A750del mutations had a median PFS of 21.2 months, shorter than patients with EGFR 19delins mutations (26.8 months), although this difference was not statistically significant (*P* = 0.258).

In addition, we compared the median PFS of the most common mutation E746_A750del (21.2 months, 95% CI: 16.3–26.1) with other uncommon mutations ranking the top three (L747_P753delinsS, L747_T751delinsP, and L747_A750delinsP). Throughout the entire follow-up, the median PFS was 38.7 months (95% CI: 0-77.4) (*P* = 0.506) for the L747_P753delinsS group, and 26.8 months (95% CI: not estimable) (*P* = 0.129) for the L747_T751delinsP group. While for the L747_A750delinsP group, it was 9.8 months (95% CI: 1.8–17.8), shorter than the E746_A750del group (*P* = 0.100).

As shown in Table 2, in the univariate analysis, patients with a family history of cancer, 4 or more metastatic sites at baseline, and bone or liver metastasis had a higher risk of disease progression, with hazard ratios (HR) of 2.62 (95% CI: 1.34–5.14, *P* = 0.005), 4.5 (95% CI: 2.13–9.51, *P* < 0.001), 2.19 (95% CI: 1.19–4.04, *P* = 0.012), and 2.57 (95% CI: 1.22–5.41, *P* = 0.013), respectively. However, mutation frequencies, the deletion start codon for the EGFR 19del, and the number of deleted nucleotides showed no significant association with the risk of progression, with HRs being 0.82 (95% CI: 0.45–1.49, *P* = 0.508), 0.67 (95% CI: 0.34–1.31, *P* = 0.238), and 0.87 (95% CI: 0.45–1.66, *P* = 0.662), respectively. Variables with P-values less than 0.1 in the univariate analysis and mutation types were included in the multivariate analysis, but no variables were identified as significantly correlated with the risk of disease progression.

**Table 2:** Univariate and multivariate Cox regression analysis of progression-free survival

Characteristics	Univariate analysis		Multivariate analysis	
	HR [95% CI]	*P* value	HR [95% CI]	*P* value
Age (≤ 65 years vs. >65 years)	1.11 [0.59–2.06]	0.752		
Gender (female vs. male)	1.52 [0.84–2.74]	0.167		
ECOG (0–1 vs. 2–3)	1.62 [0.38–6.81]	0.513		
Smoking history (no vs. yes)	1.43 [0.76–2.7]	0.271		
Tumor history (no vs. yes)	1.14 [0.35–3.7]	0.828		
Family history of tumor (no vs. yes)	2.62 [1.34–5.14]	0.005	1.86 [0.86,4.02]	0.117
Histological types (adenocarcinoma vs. others)	0.33 [0.05–2.47]	0.281		
Stage (Ⅲb-Ⅲc vs. Ⅳa-Ⅳb)	1.46 [0.52–4.1]	0.475		
Number of metastatic sites (0–3 vs. ≥4)	4.5 [2.13–9.51]	0.000	3.17 [0.68,14.92]	0.144
Bone metastasis (no vs. yes)	2.19 [1.19–4.04]	0.012	1.53 [0.77,3.04]	0.222
Liver metastasis (no vs. yes)	2.57 [1.22–5.41]	0.013	0.92 [0.23,3.69]	0.906
Brain metastasis (no vs. yes)	1.82 [0.96–3.47]	0.067	1.01 [0.46,2.22]	0.976
E746_A750del vs. others	0.82 [0.45–1.49]	0.508	1.26 [0.39,4.03]	0.7
E746 vs. L747	0.67 [0.34–1.31]	0.238	0.67 [0.17,2.6]	0.558
15n-del vs. non-15n-del	0.87 [0.45–1.66]	0.662	0.83 [0.23,2.95]	0.771

*ECOG *Eastern cooperative oncology group, *E746 *Deletions starting at E746, *L747 *Deletions starting at L747, *15n-del* Deletions of 15 nucleotides, *non-15n-del* non-15 nucleotide deletion

## Discussion

To our knowledge, this study represents the largest multicenter real-world investigation to date evaluating the impact of different EGFR 19del subtypes on the efficacy of first-line osimertinib in patients with EGFR-mutant NSCLC. In this study, the prevailing mutation in EGFR 19del was E746_A750del, followed by L747_P753delinsS, L747_T751delinsP, and L747_A750delinsP. EGFR 19del was classified according to three different patterns: common mutation (E746_A750del) or uncommon subtypes, the deletion start codon for EGFR 19del (E746 or L747), and the number of deleted nucleotides (15n-del or non-15n-del). In the present study, no statistically significant differences in treatment outcomes were observed across the major EGFR 19del subtypes. However, it is noteworthy that certain subtypes, particularly L747_A750delinsP, demonstrated a trend toward inferior PFS compared with the common E746_A750del subtype.

To date, multiple studies have investigated the impact of different EGFR 19del subtypes on EGFR-TKI efficacy using various classification strategies. Some studies grouped patients by common E746_A750del versus other variants; while Peng et al. reported significantly shorter PFS with first-generation EGFR-TKIs in the common mutation group compared with those with EGFR 19delins mutations, others found no significant difference [14–16, 18, 22]. Regarding the initial deletion site (E746 vs. L747), some studies reported longer PFS or OS with E746-initiated deletions, whereas others observed no significant difference [6, 14, 17–20]. Studies based on deletion length (15n-del vs. non-15n-del) have also yielded conflicting results regarding PFS and OS [10, 18, 22]. Collectively, these inconsistent findings highlight the ongoing controversy and underscore the need for further investigation, particularly with newer-generation TKIs.

In contrast to the extensive research on first- and second-generation EGFR-TKIs, data on third-generation TKIs such as osimertinib remain limited. Besides, the conclusions drawn from existing studies varied. Osimertinib, the third-generation EGFR-TKI, characterized by irreversible binding and high selectivity for EGFR, has demonstrated improved clinical outcomes compared with earlier-generation TKIs [27–29]. Limited studies either focused on osimertinib as second-line treatment, had insufficient sample sizes, or exclusively targeted specific subtypes of rare mutations, lacking larger sample cohorts to explore the efficacy of first-line osimertinib in various EGFR 19del subtypes.

For example, disparities in findings emerged from research like that of Peng et al., where patients with E746_A750del mutations exhibited significantly prolonged median PFS with second-line osimertinib compared to those with EGFR 19delins mutations (12 months vs. 5 months; *P* < 0.001) [15]. Conversely, Wu et al. argued that patients with different EGFR exon 19 deletion mutation subtypes showed no significant variation in PFS with second-line third-generation EGFR-TKI treatment (*P* = 0.957) [16]. Lu et al.‘s exploration of first-line osimertinib efficacy, limited to 10 patients with EGFR 19delins, resulted in a median PFS of only 6.9 months [25]. Grant et al.‘s focus on the specific L747_A750delinsP subtype found a median PFS of 11.7 months, notably shorter than patients with E746_A750del mutations at 21.3 months (*P* = 0.043) [13].

Our multicenter retrospective study included 106 patients with EGFR 19del mutations receiving first-line osimertinib. Using multiple classification methods, we observed no significant differences in osimertinib efficacy across different EGFR 19del subtypes. Notably, patients with the common E746_A750del mutation showed a numerically shorter median PFS compared to those with EGFR 19delins mutations (21.2 vs. 26.8 months, *P* = 0.258). Further analysis of 19delins subtypes revealed heterogeneous outcomes: while the L747_P753delinsS and L747_T751delinsP groups exhibited a trend toward longer PFS relative to E746_A750del, the L747_A750delinsP subgroup demonstrated a markedly shorter median PFS of only 9.8 months versus 21.2 months (*P* = 0.100). Importantly, this finding aligns with previous reports suggesting that specific EGFR 19del subtypes—particularly L747_A750delinsP—may exhibit reduced sensitivity to osimertinib [13, 30, 31].

Our study has several limitations. First, due to its retrospective design and relatively limited sample size, caution is warranted when generalizing the findings. Second, because osimertinib was only recently approved in 2019 for first-line treatment of advanced NSCLC patients with EGFR-sensitive mutations in China, the follow-up duration remains relatively short. As a result, the median PFS for certain subgroups has not yet been reached, and OS data are still immature; therefore, OS analysis was not performed in the present study. Third, this study focused primarily on the impact of EGFR 19del subtypes on treatment efficacy and did not incorporate a comprehensive analysis of co-occurring genomic alterations. Given the emerging evidence that co-mutations may influence responses to EGFR-TKIs, this represents an important area for further investigation. Future studies with longer follow-up, larger cohort sizes, and integrated analyses of co-mutation profiles are warranted to more precisely characterize the heterogeneity of EGFR 19del subtypes and their impact on osimertinib efficacy.

## Conclusions

In conclusion, this study found no statistically significant associations between the efficacy of first-line osimertinib and different classification patterns of EGFR 19del, including mutation frequency (common E746_A750del vs. uncommon subtypes), deletion start codon (E746 vs. L747), and the number of deleted nucleotides (15n-del vs. non-15n-del). However, a trend toward heterogeneity was observed among specific mutation subtypes. In particular, the L747_A750delinsP subtype demonstrated a numerically shorter PFS, suggesting that certain EGFR 19del variants may exhibit attenuated sensitivity to osimertinib. Taken together, these findings indicate that, while overall efficacy appears broadly comparable across EGFR 19del subtypes, potential subtype-specific variability should not be overlooked. Further studies with larger cohorts, longer follow-up, and integrated molecular analyses are warranted to better elucidate the clinical significance of this heterogeneity in EGFR-mutant NSCLC.

## Data Availability

The datasets generated and analysed during the current study are available in the Zenodo repository at https://doi.org/10.5281/zenodo.17694242.

## Abbreviations

CI: Confidence Interval
COSMIC: Catalogue of Somatic Mutations in Cancer
CR: Complete Response
ECOG: Eastern Cooperative Oncology Group
EGFR: Epidermal Growth Factor Receptor
HR: Hazard Ratio
NGS: Next-Generation Sequencing
NSCLC: Non-Small Cell Lung Cancer
ORR: Objective Response Rate
PD: Progressive Disease
PFS: Progression-Free Survival
PR: Partial Response
RECIST: The Response Evaluation Criteria in Solid Tumours
SD: Stable Disease
TKIs: Tyrosine Kinase Inhibitors

## References

[1] da Cunha Santos G, Shepherd FA, Tsao MS. EGFR mutations and lung cancer. Annu Rev Pathol. 2011;6:49–69. 20887192 10.1146/annurev-pathol-011110-130206

[2] Meng H, Guo X, Sun D, Liang Y, Lang J, Han Y, et al. Genomic Profiling of Driver Gene Mutations in Chinese Patients With Non-Small Cell Lung Cancer. Front Genet. 2019;10:1008. 31749831 10.3389/fgene.2019.01008 PMC6842958

[3] Wang H, Huang J, Yu X, Han S, Yan X, Sun S, et al. Different efficacy of EGFR tyrosine kinase inhibitors and prognosis in patients with subtypes of EGFR-mutated advanced non-small cell lung cancer: a meta-analysis. J Cancer Res Clin Oncol. 2014;140(11):1901–9. 24908327 10.1007/s00432-014-1709-0 PMC4196046

[4] Lee CK, Wu YL, Ding PN, Lord SJ, Inoue A, Zhou C, et al. Impact of Specific Epidermal Growth Factor Receptor (EGFR) Mutations and Clinical Characteristics on Outcomes After Treatment With EGFR Tyrosine Kinase Inhibitors Versus Chemotherapy in EGFR-Mutant Lung Cancer: A Meta-Analysis. J Clin Oncol. 2015;33(17):1958–65. 25897154 10.1200/JCO.2014.58.1736

[5] Castellanos E, Feld E, Horn L. Driven by Mutations: The Predictive Value of Mutation Subtype in EGFR-Mutated Non-Small Cell Lung Cancer. J Thorac Oncol. 2017;12(4):612–23. 28017789 10.1016/j.jtho.2016.12.014

[6] Lee VH, Tin VP, Choy TS, Lam KO, Choi CW, Chung LP, et al. Association of exon 19 and 21 EGFR mutation patterns with treatment outcome after first-line tyrosine kinase inhibitor in metastatic non-small-cell lung cancer. J Thorac Oncol. 2013;8(9):1148–55. 10.1097/JTO.0b013e31829f684a 23945384

[7] Soria JC, Ohe Y, Vansteenkiste J, Reungwetwattana T, Chewaskulyong B, Lee KH, et al. Osimertinib in Untreated EGFR-Mutated Advanced Non-Small-Cell Lung Cancer. N Engl J Med. 2018;378(2):113–25. 29151359 10.1056/NEJMoa1713137

[8] He C, Wei C, Wen J, Chen S, Chen L, Wu Y, et al. Comprehensive analysis of NGS and ARMS-PCR for detecting EGFR mutations based on 4467 cases of NSCLC patients. J Cancer Res Clin Oncol. 2022;148(2):321–30. 34693477 10.1007/s00432-021-03818-w PMC8800890

[9] Tate JG, Bamford S, Jubb HC, Sondka Z, Beare DM, Bindal N, et al. COSMIC: the Catalogue Of Somatic Mutations In Cancer. Nucleic Acids Res. 2019;47(D1):D941–7. 30371878 10.1093/nar/gky1015 PMC6323903

[10] Tokudome N, Koh Y, Akamatsu H, Fujimoto D, Okamoto I, Nakagawa K, et al. Differential significance of molecular subtypes which were classified into EGFR exon 19 deletion on the first line afatinib monotherapy. BMC Cancer. 2020;20(1):103. 32028909 10.1186/s12885-020-6593-1 PMC7006223

[11] Wang Y, Zheng R, Hu P, Zhang Z, Shen S, Li X. Patients harboring uncommon EGFR exon 19 deletion-insertion mutations respond well to first-generation EGFR inhibitors and osimeritinib upon acquisition of T790M. BMC Cancer. 2021;21(1):1215. 34774017 10.1186/s12885-021-08942-x PMC8590339

[12] Chung KP, Wu SG, Wu JY, Yang JC, Yu CJ, Wei PF, et al. Clinical outcomes in non-small cell lung cancers harboring different exon 19 deletions in EGFR. Clin Cancer Res. 2012;18(12):3470–7. 22510346 10.1158/1078-0432.CCR-11-2353

[13] Grant MJ, Aredo JV, Starrett JH, Stockhammer P, van Alderwerelt IK, Wurtz A, et al. Efficacy of Osimertinib in Patients with Lung Cancer Positive for Uncommon EGFR Exon 19 Deletion Mutations. Clin Cancer Res. 2023;29(11):2123–30. 36913537 10.1158/1078-0432.CCR-22-3497 PMC10493186

[14] Rossi S, Toschi L, Finocchiaro G, Di Noia V, Bonomi M, Cerchiaro E, et al. Impact of Exon 19 Deletion Subtypes in EGFR-Mutant Metastatic Non-Small-Cell Lung Cancer Treated With First-Line Tyrosine Kinase Inhibitors. Clin Lung Cancer. 2019;20(2):82–7. 30473385 10.1016/j.cllc.2018.10.009

[15] Peng X, Long X, Liu L, Zeng L, Yang H, Jiang W, et al. Clinical impact of uncommon epidermal growth factor receptor exon 19 insertion-deletion variants on epidermal growth factor receptor-tyrosine kinase inhibitor efficacy in non-small-cell lung cancer. Eur J Cancer. 2020;141:199–208. 33171317 10.1016/j.ejca.2020.10.005

[16] Wu SG, Gow CH, Chen YL, Liu YN, Tsai MF, Shih JY. Different treatment efficacies and T790M acquisition of EGFR-TKIs on NSCLC patients with variable Del-19 subtypes of EGFR. Int J Cancer. 2023;153(2):352–63. 36912241 10.1002/ijc.34507

[17] Sutiman N, Tan SW, Tan EH, Lim WT, Kanesvaran R, Ng QS, et al. EGFR Mutation Subtypes Influence Survival Outcomes following First-Line Gefitinib Therapy in Advanced Asian NSCLC Patients. J Thorac Oncol. 2017;12(3):529–38. 27908825 10.1016/j.jtho.2016.11.2225

[18] Huang LT, Zhang SL, Han CB, Ma JT. Impact of EGFR exon 19 deletion subtypes on clinical outcomes in EGFR-TKI-Treated advanced non-small-cell lung cancer. Lung Cancer. 2022;166:9–16. 35151115 10.1016/j.lungcan.2022.01.014

[19] Kaneda T, Hata A, Tomioka H, Tanaka K, Kaji R, Fujita S, et al. Possible differential EGFR-TKI efficacy among exon 19 deletional locations in EGFR-mutant non-small cell lung cancer. Lung Cancer. 2014;86(2):213–8. 25304185 10.1016/j.lungcan.2014.09.014

[20] Su J, Zhong W, Zhang X, Huang Y, Yan H, Yang J, et al. Molecular characteristics and clinical outcomes of EGFR exon 19 indel subtypes to EGFR TKIs in NSCLC patients. Oncotarget. 2017;8(67):111246–57. 29340050 10.18632/oncotarget.22768 PMC5762318

[21] Xu H, Li W, Yang G, Li J, Yang L, Xu F, et al. Heterogeneous Response to First-Generation Tyrosine Kinase Inhibitors in Non-Small-Cell Lung Cancers with Different EGFR Exon 19 Mutations. Target Oncol. 2020;15(3):357–64. 32418166 10.1007/s11523-020-00722-0

[22] Gu Y, Yu J, Hu H, Zhang H, Cao B, Liang L. Clinical outcomes of advanced NSCLC patients with different EGFR exon 19 deletion subtypes treated with first-line tyrosine kinase inhibitors: A single-center ambispective cohort study. Thorac Cancer. 2023;14(31):3147–60. 37704565 10.1111/1759-7714.15108 PMC10626247

[23] Chen Y, Xu J, Zhang L, Song Y, Wen W, Lu J, et al. A multicenter-retrospective study of non-small-cell lung carcinoma harboring uncommon epidermal growth factor receptor (EGFR) mutations: different subtypes of EGFR exon 19 deletion-insertions exhibit the clinical characteristics and prognosis of non-small cell lung carcinoma. Transl Lung Cancer Res. 2022;11(2):238–49. 35280318 10.21037/tlcr-22-48 PMC8902088

[24] Zhao C, Jiang T, Li J, Wang Y, Su C, Chen X, et al. The impact of EGFR exon 19 deletion subtypes on clinical outcomes in non-small cell lung cancer. Transl Lung Cancer Res. 2020;9(4):1149–58. 32953493 10.21037/tlcr-19-359 PMC7481579

[25] Lu Z, Yi Y, Wang L, Luo Y, Luo D, Xiong L, et al. Non-small cell lung cancer cells with uncommon EGFR exon 19delins variants respond poorly to third-generation EGFR inhibitors. Transl Oncol. 2024;39:101834. 38006760 10.1016/j.tranon.2023.101834 PMC10728704

[26] Xu Y, Zhang L, Fang J, Wang Z, Li J, Li L, et al. Establishment of a prospective multicenter cohort for advanced non-small cell lung cancer in China (CAPTRA-Lung study). Thorac Cancer. 2018;9(12):1795–800. 30264504 10.1111/1759-7714.12865 PMC6275840

[27] Starrett JH, Guernet AA, Cuomo ME, Poels KE, van Alderwerelt IK, Nagelberg A, et al. Drug Sensitivity and Allele Specificity of First-Line Osimertinib Resistance EGFR Mutations. Cancer Res. 2020;80(10):2017–30. 32193290 10.1158/0008-5472.CAN-19-3819 PMC7392201

[28] Zhang D, Liu X, Shen F, Zhao D, Shi Y, Zhang H, et al. Osimertinib versus comparator first-generation epidermal growth factor receptor tyrosine kinase inhibitors as first-line treatment in patients with advanced EGFR-mutated non-small cell lung cancer: a Chinese, multicenter, real-world cohort study. Transl Lung Cancer Res. 2023;12(11):2229–44. 38090527 10.21037/tlcr-23-577 PMC10713260

[29] Ramalingam SS, Vansteenkiste J, Planchard D, Cho BC, Gray JE, Ohe Y, et al. Overall Survival with Osimertinib in Untreated, EGFR-Mutated Advanced NSCLC. N Engl J Med. 2020;382(1):41–50. 31751012 10.1056/NEJMoa1913662

[30] Truini A, Starrett JH, Stewart T, Ashtekar K, Walther Z, Wurtz A, et al. The EGFR Exon 19 Mutant L747-A750 > P Exhibits Distinct Sensitivity to Tyrosine Kinase Inhibitors in Lung Adenocarcinoma. Clin Cancer Res. 2019;25(21):6382–91. 31182434 10.1158/1078-0432.CCR-19-0780 PMC6825535

[31] Brown BP, Zhang YK, Kim S, Finneran P, Yan Y, Du Z, et al. Allele-specific activation, enzyme kinetics, and inhibitor sensitivities of EGFR exon 19 deletion mutations in lung cancer. Proc Natl Acad Sci U S A. 2022;119(30):e2206588119. 35867821 10.1073/pnas.2206588119 PMC9335329

